# *Penicillium setosum*, a new species from *Withania somnifera* (L.) Dunal

**DOI:** 10.1080/21501203.2018.1555868

**Published:** 2018-12-12

**Authors:** Tijith K. George, Jos Houbraken, Linu Mathew, M. S. Jisha

**Affiliations:** a School of Biosciences, Mahatma Gandhi University, Kottayam, India; b Westerdijk Fungal Biodiversity Institute, Utrecht, The Netherlands

**Keywords:** Ascospores, Conidiophores, Endophyte, *Penicillium javanicum-*clade, Molecular phylogeny

## Abstract

Medicinal plants are considered as sources of novel and unexplored groups of endophytic microorganisms. A study on endophytic fungal species from the medicinal plant *Withania somnifera* (L.) Dunal resulted in the isolation of a *Penicillium* isolate (WSR 62) with antibiotic activity. Phylogenetic analysis showed that the isolate belongs to section *Lanata-divaricata*, and it is most closely related to *P. javanicum*. Subsequent detailed phylogenetic analyses using partial β-tubulin (*BenA*), calmodulin (*CaM*) and DNA-dependent RNA polymerase II (*RPB2*) gene sequences of a larger number of related strains revealed the distinctiveness of the isolate in the *P. javanicum-*clade. The isolate grows fast on Czapek yeast autolysate agar (CYA) and malt extract agar (MEA) incubated at 25°C, 30°C and 37°C. The obverse colony colour is dominated by the conspicuous production of cleistothecia and is greyish yellow on CYA and yellowish brown on MEA. Production of cleistothecia containing prominent spinose ascospores was present on all tested agar media. Based on the phylogenetic analysis and the phenotypic characterisation, strain WSR 62 from *Withania* is described here as a novel species named *Penicillium setosum.*

## 1. Introduction

Endophytes are microorganisms living within a plant throughout its life cycle or complete at least a part of its life cycle without causing any apparent disease in the host plant (Petrini 1991). They are ubiquitous and can be involved in neutral, commensal and/or beneficial associations with the host plant and tend to become opportunistic saprobes or pathogens at a later phase of their life cycle (Compant et al. 2016). The endophytic mode of life helps them to enhance their dispersal and attain suitable substrates and environmental conditions for their survival (Thomas et al. 2017). Their associations with their host plant enable them to establish a microbial community in the plant endosphere (Hardoim et al. 2015). Such colonisation of endophytes offers a unique biological niche favouring their growth and grants them a distinctive ability to produce new bioactive metabolites. Hence, exhaustive searches are being made for discovering their diversity and usefulness in agriculture, environment and medicine (Jia et al. 2016).

*Penicillium* is one of the most common and diverse fungal genera, has a worldwide distribution and contains both beneficial and harmful members. This genus currently includes 429 accepted species (Visagie et al. 2014, www.aspergilluspenicillium.org). Species of this economically important genus are commercial sources of antibiotics, organic acids, alcohols, enzymes, pharmaceuticals, and other metabolites (Frisvad et al. 2004). Nonetheless, some of them cause food spoilage, produce mycotoxins, and cause human and animal diseases (Pitt 1994). The predominance of *Penicillium* in nature is due to their ability to thrive in multiple ecological niches such as soil, decomposing organic materials, food and feed, and marine environment. Some species of *Penicillium* follow an endophytic mode of existence and can reside on, e.g. rhizosphere, laminosphere, and phyllosphere. *Penicillium* features a prominent position among the culturable endophytic microbes (Potshangbam et al. 2017). For example, *Penicillium* cf. *glaucoalbidum* from section *Thysanophora* (previously the genus *Thysanophora*) is often isolated from *Picea rubens* (Sieber-Canavesi & Sieber 1993; Müller & Hallaksela 1998; Koukol et al. 2012). Some other reports for the endophytic occurrence of this genus are *Penicillium coffeae* from *Coffea arabica* (Peterson et al. 2005), *P. citrinum* from *Ixeris repenes* (Khan et al. 2008), *P. goetzii* from *Pinus ponderosa* and *Pseudotsuga menziesii* (Houbraken et al. 2012), *P. glabrum* from *Punica granatum* (Hammerschmidt et al. 2012), and *P. fluviserpens* from coffee plant (Peterson et al. 2015). Mechanisms behind invasion, establishment, endophytic association and also beneficial role or harmfulness of this genus are not yet clear (Compant et al. 2016).

*Withania somnifera* (common name: Ashwagandha, Family: Solanaceae) is a well-known medicinal plant found naturally in arid wastelands of the tropics. It is a perennial shrub growing up to 30 cm to 1.5 m in height. The whole plant including root, leaves, and berries has been used in the Ayurveda system of indigenous medicine for centuries as a *Rasayana* herb (Ven Murthy et al. 2010). Many bioactive substances are extracted from this plant and used for their immunomodulatory, anti-stress and anti-tumour activity (Bharti et al. 2016). The ayurvedic formulations of this plant exhibit multiple pharmacological actions, like general tonic, sedative, liver tonic and anti-inflammatory activity. Studies also showed that leaves and roots of Ashwagandha have antibacterial properties (Owais et al. 2005). Nevertheless, only scanty reports are available regarding the endophytes present in this plant.

In our search for endophytic fungi from *W. somnifera*, with the ability to produce antimicrobial compounds, we encountered a novel *Penicillium* species. The present study aims to describe this endophytic *Penicillium* isolate from the surface-sterilized roots of *W. somnifera* based on phenotypic and phylogenetic analysis.

## 2. Materials and methods

### 2.1. Collection of *Withania somnifera*

**Figure 1.**
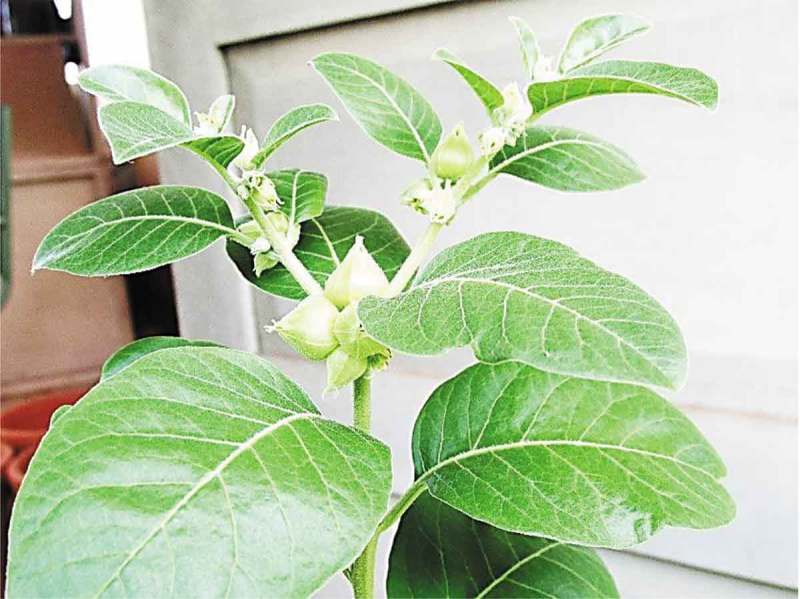
*Withania somnifera* (L.) Dunal.

*Withania somnifera* (L.) Dunal (Figure 1) plantlets were collected from the nursery of Aromatic and Medicinal Plants Research Station (Kerala Agricultural University), Odakali, Ernakulam District, Kerala, India and a voucher specimen, RHK 6350, was deposited at the Regional Herbarium of Kerala, St. Berchman’s College, Kerala, India.

### 2.2. Isolation

Healthy, fully grown plants were used for the isolation of the endophytes. Plants were uprooted, brought to the laboratory in sealed sterile polythene bags and processed immediately. Surface sterilization of plant roots was performed according to a modified method of George et al. (2015). Plant parts were washed in running tap water for 10 min followed by washing with Tween 40 (1%) solution. The detergent was completely removed by washing under running tap water. Thereafter, sequential rinsing of the plant material was done with 70% ethanol for 1 min, 4% sodium hypochlorite (NaOCl) for 2–3 min and again with 70% ethanol for 30 s. Final rinses were made with three changes of sterile distilled water, with total wash duration of 3 min. Successful surface sterilization was ensured by pressing randomly chosen plant parts onto culture plates for a few seconds and looking for any microbial growth for a period of 2 weeks. Surface-sterilised plant roots were cut into small pieces (≈1 cm) and each piece was placed on potato dextrose agar (PDA), malt extract agar (MEA) and yeast extract glucose agar (YEGA) supplemented with streptomycin (3 mg/100 mL) (HiMedia laboratories, Mumbai, India). Plates were incubated at 26 ± 2°C, till the appearance of fungal mycelia (2–3 weeks). A total of 73 fungal isolates were obtained. They were purified, inoculated onto PDA slants and kept as stock cultures at 4°C. Among these isolates, a *Penicillium* isolate (WSR 62) with antibacterial activity was studied in detail.

### 2.3. DNA extraction, sequencing and phylogenetic analysis

The total genomic DNA of WSR 62 grown in potato dextrose broth was isolated by using the fungal genomic DNA minispin kit RKN 19 (Chromous Biotech, India). The internal transcribed spacer (ITS) was amplified from the isolated fungal genomic DNA using the universal ITS primers (ITS1 and ITS4) (White et al. 1990). Additionally, partial β-tubulin (*BenA*), calmodulin (*CaM*) and RNA polymerase II second largest subunit (regions 5–7) (*RPB2*) genes were amplified, using the PCR conditions and primers as described by Visagie et al. (2014). PCR reaction volumes of 50 µL contained 2 µL (10 pmol/µL) of each primer, 5 µL of DNA template (30 ng) and 25 µL of 2X OrionX Taq PCR smart mix (Origin, India). Amplification was carried out in a Bio-Rad thermocycler (Bio-Rad, USA) programmed with an initial denaturation at 95°C for 5 min, followed by 30 cycles of 94°C for 30 s, 55°C for 30 s and 72°C for 45 s, with a final extension at 72°C for 10 min. The PCR product was electrophoresed on 1.2% agarose gel in 0.5X TBE buffer at 65 V for 1 h to check the amplicons. The Illustra GFX PCR DNA and gel band purification kit (GE Healthcare, USA) was used for the rapid purification and concentration of PCR products. Sequencing of the amplicons was done by Big dye terminator v3.2 cycle sequencing chemistry for ABI Bioprism (Applied Biosystems, USA). Forward and reverse strands of all four genomic regions were sequenced, reverse strands were reverse complemented, aligned by BioEdit (Hall 1999) and analysed manually for any errors. The sequences were queried using the BLAST (www.ncbi.nlm.nih.gov) search algorithm and deposited in the NCBI GenBank (ITS- KT852579, *BenA*- MF184995, *CaM*- MH105905 and *RPB2*- MH016196).

The phylogenetic position of isolate WSR 62 in section *Lanata-divaricata* was studied using a combined data set of ITS, *BenA* and *CaM* sequences. The generated sequences of isolate WSR 62 were compared with a reference data set containing sequences of the type strains belonging to section *Lanata-divaricata* (Houbraken et al. 2011; Visagie et al. 2014, 2015) (Supplementary Table 1). Sequence alignments were performed using the CLUSTAL W program in BioEdit and alignments were manually optimised. The Akaike information criterion (AIC) in the program jModelTest version 2.1.5 (Darriba et al. 2012) was employed to determine the most optimal substitution model. *Penicillium glabrum* CBS 125543^T^ was used as outgroup. Data sets were analysed using MrBayes software v. 3.2.2 (Ronquist et al. 2012) by running 200,000 generations of two independent chains. At this point, the split frequencies fell below 0.05. The Bayesian posterior probability (pp) values were used to determine the node reliability. Trees were visualised in FigTree version 1.4.3 (Rambaut 2016). Maximum Likelihood (ML) analysis was performed using RaxML (Randomized Accelerated Maximum Likelihood) v 7.2.5 and the bootstrap support of the nodes was determined. The obtained bootstrap values are plotted on the Bayesian phylogram. In order to capture the intraspecies variation of *P. javanicum* in this study, the generated *BenA, CaM*, and *RPB2* sequences of WSR 62 were compared with sequences derived from strains maintained as *P. javanicum* in the CBS culture collection or the internal working collection of the department Applied and Industrial Mycology (DTO) housed at the Westerdijk Fungal Biodiversity Institute. An overview of studied strains is given in Table 1. The single gene and combined data sets were aligned and Maximum Likelihood analysis was performed as described by Frisvad et al. (2019). Sequences of *P. oxalicum* CBS 219.30^T^ were used as outgroup. The alignments were submitted to TreeBase with submission ID 22415.

**Table 1.** Strains used in the phylogenetic analyses of the *Penicillium javanicum* clade.

Species name	Strain number	Substrate and location
*P. caperatum*	CBS 443.75 = ATCC 28046 = DSM 2209	Soil, Western Highlands, Baiyer River, Papua New Guinea; type of *P. caperatum*
*P. elleniae*	CBS 118135 = IBT 23229	Leaf litter in litter bag exposed for 6 months, Araracuara, Colombia; type of *P. elleniae*
*P. javanicum*	CBS 127811 = DTO 108-E2 = IBT 4446 = NRRL 2078	Soil, Brazil
*P. javanicum*	CBS 251.66 = DTO 097-D2	Savannah soil, Abidjan, Ivory Coast
*P. javanicum*	CBS 341.48 = DTO 097-F9	Root of *Camellia sinensis*, Buitenzorg, Java, Indonesia; type of *P. javanicum* and *P. indonesiae*
*P. javanicum*	CBS 349.51 = DTO 097-F8	Unknown source, Japan; type of *P. oligosporum*
*P. javanicum*	DTO 010-C3	Soil from citrus grove, Kissimmee near Lake Tohopekaliga, Florida, USA
*P. javanicum*	DTO 111-A9 = IBT 29369	Soil, Campinas, Brazil
*P. javanicum*	DTO 344-A1	Soil, La Reunion, France
*P. malacosphaerulum*	CBS 135120 = DAOM 241161	Soil, Malmesbury, South-Africa, type of *P. malacosphaerulum*
*P. reticulisporum*	CBS 121.68 = DTO 097-C1 = ATCC 18565 = IMI 136699 = NRRL 3446	Soil, Yamanshi Pref., Kajikazawa-machi, Jukkoku, Japan; AUT of *E. reticulisporum*
*P. reticulisporum*	CBS 122.68 = ATCC 18566 = IMI 136700 = NRRL 3447	Soil, Oshima Islands, Molo-machi, Japan; type of *P. reticulisporum*
*P. reticulisporum*	CBS 123.68	Soil, Tokyo, Oshima Isl., Moto-machi, Japan; AUT of *E. reticulisporum*
*P. reticulisporum*	CBS 513.74 = DTO 095-E2 = DSM 2207	Soil, Kôchi Pref., Takaoka-gun, Ochi-machi, Japan; type of *E. arvense*
*P. setosum*	CBS 576.70 = DTO 096-I2	Soil, Nayarit State, 30 km N of San Blas, Mexico
*P. setosum*	DTO 284-F3	Soil from corn field, Thailand
*P. setosum*	WSR 62 = CBS 144865 = MCC 1370	Endophyte from *Withania somnifera*, India; type of *P. setosum*
*P. uruguayense*	CBS 143247 = FMR 14490	Soil, Colonia del Sacramento; Uruguay; type of *P. uruguayense*
*Penicillium* sp.	CBS 564.85 = DTO 097-C2	Culture contaminant from CBS 497.85; Toronto, Canada

### 2.4. Morphology

The colony descriptions of isolate WSR 62 were based on growth on MEA (media composition: malt extract – 50 gL^−1^ (HiMedia, India), trace elements stock solution – 1 mLL^−1^ (trace elements stock solution (100 mL): CuSO_4._5H_2_O – 0.5 g, ZuSO_4._7H_2_O – 0.1 g) (Samson et al. 2010), Czapek dox agar (CA), Czapek yeast autolysate agar (CYA), Czapek yeast autolysate agar with 5% NaCl (CYAS), and yeast extract sucrose agar (YES). The media composition, fungal inoculation procedure and incubation were performed as described in Visagie et al. (2014). After incubation, colony characters such as colony diameter, pigment production, exudate production, obverse and reverse colony colour, hyphal and mycelial growth, surface texture and degree of sporulation were recorded thrice to confirm the stability of morphological characters. Identification methods proposed by Raper and Thom (1949), Pitt (1974), Pitt (1980), Frisvad and Samson (2004) and Visagie et al. (2014) were employed for the morphological characterisation of the fungus. Ten-day old colonies from MEA were used for light microscopy and SEM imaging of cleistothecia, ascospores and conidial structures. The slides were examined using a Motic BA 310 (USA) light microscope. Conidiophore structures were measured using Motic Image Plus 2.0 ML software. Micrographs of individual cleistothecia were taken with a Labomed Lx400 microscope equipped with a Micaps digital camera run by Micaps software. To prepare the scanning electron microgram of the structures, fungal samples were fixed overnight in 2.5% glutaraldehyde at 4°C. Slides were washed in phosphate buffer (pH 7.4), followed by sequential dehydration by series of washes with acetone/alcohol (30%, 50%, 70%, 80%, 90% and 100%) (Hartmann et al. 2010). The dried material was mounted on stubs, sputtered with gold and examined with a scanning electron microscope (SEM; JSM-6490LA, JEOL, Tokyo, Japan). The ascospores and spines were measured on the SEM micrographs.

## 3. Results

### 3.1. Phylogenetic analyses

**Figure 2.**
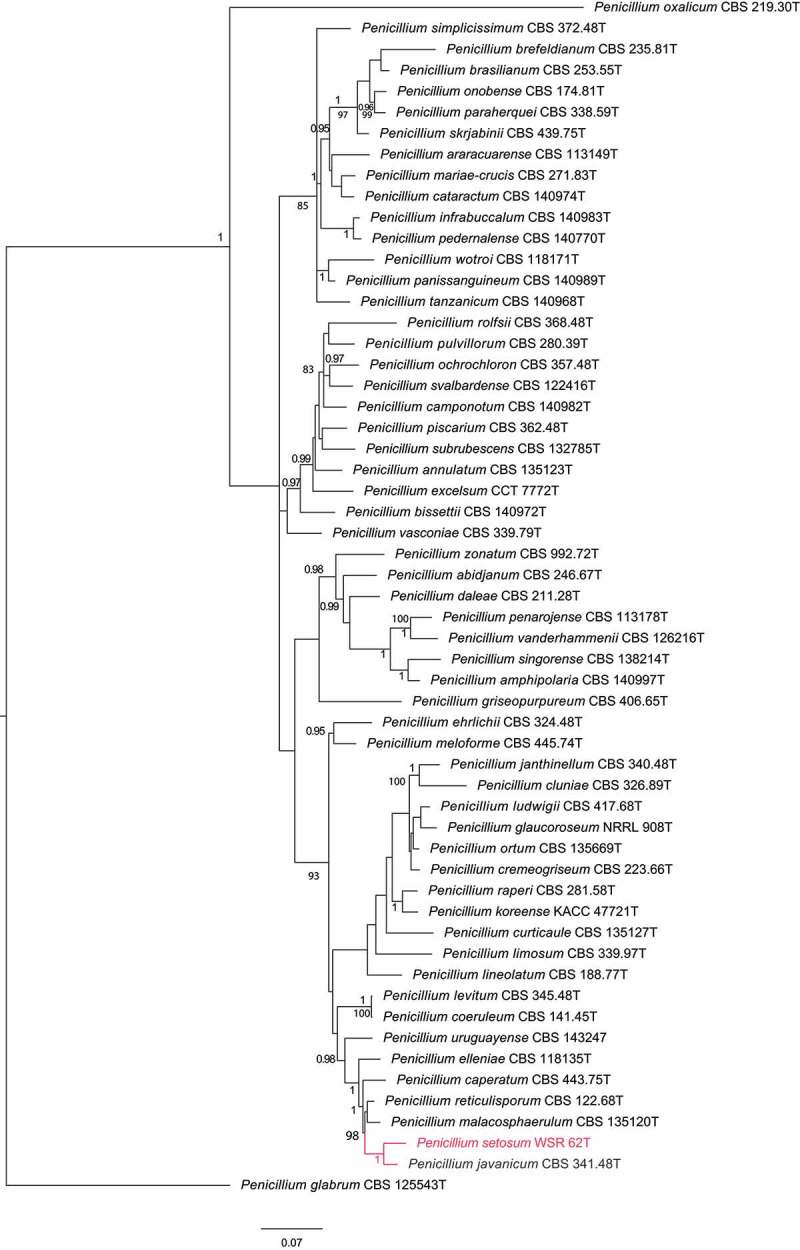
Phylogenetic tree based on a combination of partial ITS, *BenA* and *CaM* sequences of *Penicillium* sect. of *Lanata-divaricata*. The isolate *Penicillium setosum* is highlighted in red colour. *Penicillium glabrum* CBS 125543^T^ was chosen as outgroup. Only BI posterior probability values higher than 0.95 and bootstrap value above 80% are shown at the branch nodes.

**Figure 3.**
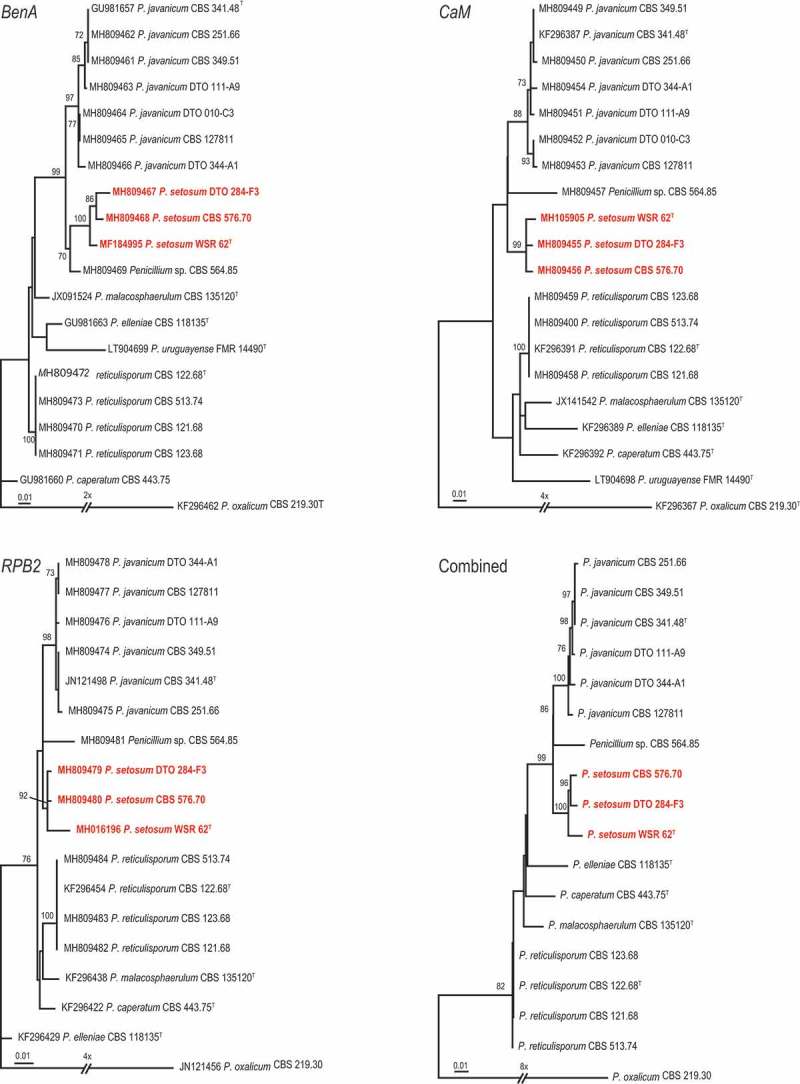
Maximum-likelihood trees based on *BenA, CaM, RPB2* data sets and the combined data sets of afore mentioned genes showing the relationship of species belonging to the *Penicillium javanicum-*clade. *Penicillium oxalicum* was chosen as outgroup in the phylogenies. Numbers at branch nodes refer to bootstrap values (1,000 replicates), only values of >70% are shown. Names in red indicate strains belonging to the new species.

The phylogenetic relationship of isolate WSR 62 was studied using a combined multilocus sequence alignment of ITS, *BenA* and *CaM* sequences of members of *Penicillium* section *Lanata-Divaricata* (Figure 2). The General Time Reversible (GTR) model using a discrete gamma distribution (+G) and invariant sites (+I) was optimal for the multilocus data set. The analysis showed that isolate WSR 62 is related to *P. javanicum, P. elleniae, P. reticulisporum, P. malacosphaerulum, P. caperatum,* and *P. urugayense*, a group of species previously referred to as the *P. javanicum*-clade (Visagie et al. 2015). Furthermore, this analysis showed that WSR 62 clustered with high statistical support (1 pp) to *P. javanicum*. The phylogenetic relationships and intraspecies variation within the *P. javanicum*-clade was studied in more detail using a larger selection of strains (Figure 3). The length of the aligned *BenA, CaM*, and *RPB2* data sets were 482, 465, and 755 bp, respectively (including gaps). The Kimura 2-parameter (K2) model using the gamma distribution (+G) was found to be optimal for the *BenA* data set, the GTR+G model was optimal for the *CaM* data set and the Tamura-Nei (TN) model using the gamma distribution (+G) was most optimal for the *RPB2* data set. The topologies of the single locus phylogenies were largely congruent, though some inconsistencies were present among the strains identified as *P. javanicum*. The strains DTO 284-F3 and CBS 576.70 clustered in all analyses with high bootstrap support (>92 %) with isolate WSR 62 and this analysis showed that these can be identified as *P. setosum* as well. CBS 564.85 is also closely related to the *P. javanicum* and *P. setosum*. The phylogenetic position of this strain is unresolved and might represent a new species in this clade.

### 3.2. Morphology and physiology

Among the isolated endophytes from *Withania somnifera*, the *Penicillium* isolate WSR 62 was studied in detail because of its antibacterial activity. The micro- and macro-morphological characters of this strain were compared to their closest relatives based on notes provided in previous studies (Pitt 1974; Stolk & Samson 1983; Visagie et al. 2014). WSR 62 grew well on MEA and CYA incubated at 25°C (CYA 50–52 mm, MEA 52–55 mm) and produced prominent spinose ascospores measuring 2.0–3.0 × 2.3–3.0 μm.

### 3.3. Taxonomy

Multigene phylogenetic analyses consistently placed isolate WSR 62 in a separate clade distinct from *P. javanicum*. This strain is phenotypically distinct from *P. javanicum* and other phylogenetically related species. Based on these data, we describe this isolate as a new species named *Penicillium setosum*.

**Figure 4.**
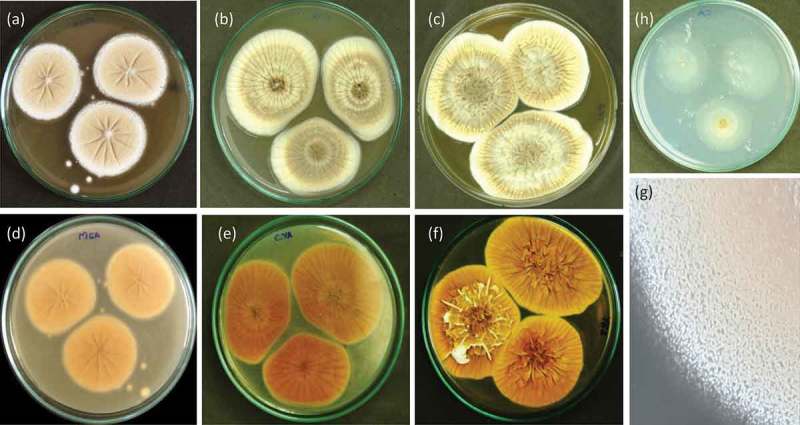
*Penicillium setosum* (WSR 62): a, b, c. Colonies on MEA, CYA and YES (all obverse); d, e, f. Colonies on MEA, CYA and YES (all reverse); g. Texture on MEA; h. Colony on CA.

***Penicillium setosum*** Tijith, Houbraken, Linu & Jisha *sp. nov*., MycoBank MB818581. Figure 4.

In: *Penicillium* subgenus *Aspergilloides*, section *Lanata-divaricata.*

*ITS barcode*: KT852579, Alternative identification markers: *BenA = *MF184995, *CaM = *MH105905, *RPB2 *= MH016196.

#### Etymology

*setosum* (Latin), bristly, referring to the surface of ascospores having prominent spinulose structure.

#### Typification

India, Kerala, isolated from *Withania somnifera* (L.) Dunal, 21 November 2014, *T. K. George* (holotype WSR 62, culture ex-type CBS 144865 = MCC 1370 = NCFT NO 8222.16 = AMH-9974).

*Colony diameter*, 7 d (in mm) of incubation: CYA (at 25°C) 50–52; CYA (at 30°C) 50–53; CYA at (37°C) 40–42; CYA with 5% NaCl (at 25°C) 50–53, MEA (at 25°C) 52–55; CA (at 25°C) 36–40, YES (at 25°C) 48–53.

#### Macromorphology

CYA 25°C, 7 d: Colonies velvety, moderately deep, radially sulcate; margins low, wide, entire; mycelia white and inconspicuously yellow; cleistothecia abundantly produced mainly near the colony centre, maturing after 1–2 weeks, giving a greyish yellow colour to colony; sporulation absent or sparse; soluble pigments absent; exudates present as small hyaline to yellow droplets; reverse orange-brown. MEA 25°C, 7 d: Colonies granular, plane; margins low, wide, entire; mycelia yellowish white; cleistothecia abundantly produced in the centre, maturing within 1–2 weeks, giving an yellowish brown colour to colony; sporulation sparse; soluble pigments absent; exudates absent; reverse yellowish orange. YES 25°C, 7 d: Colonies deep, cerebriform at the centre, radially and concentrically sulcate, raised at centre; margins low, wide, entire; mycelia white turning slightly yellow in time; cleistothecia abundantly produced, maturing within 2 weeks, giving an yellow colour to colony; sporulation absent; soluble pigments absent; exudates present as small yellow droplets; reverse olive brown. CYAS 25°C, 7 d: Colonies moderately deep; margins low, wide, entire; mycelia white, slightly mauve in the centre; texture floccose to velutinous; cleistothecia produced at the centre, giving a yellowish brown colour to colony; sporulation absent; soluble pigments absent; exudates absent; reverse olive orange. CA 25°C, 7 d: Colonies plane, thin; margins low, narrow, entire; mycelia greyish white; sporulation absent; cleistothecia moderately produced, giving a yellowish colour to colony; soluble pigments absent; exudates absent; reverse whitish grey.

#### Micromorphology

**Figure 5.**
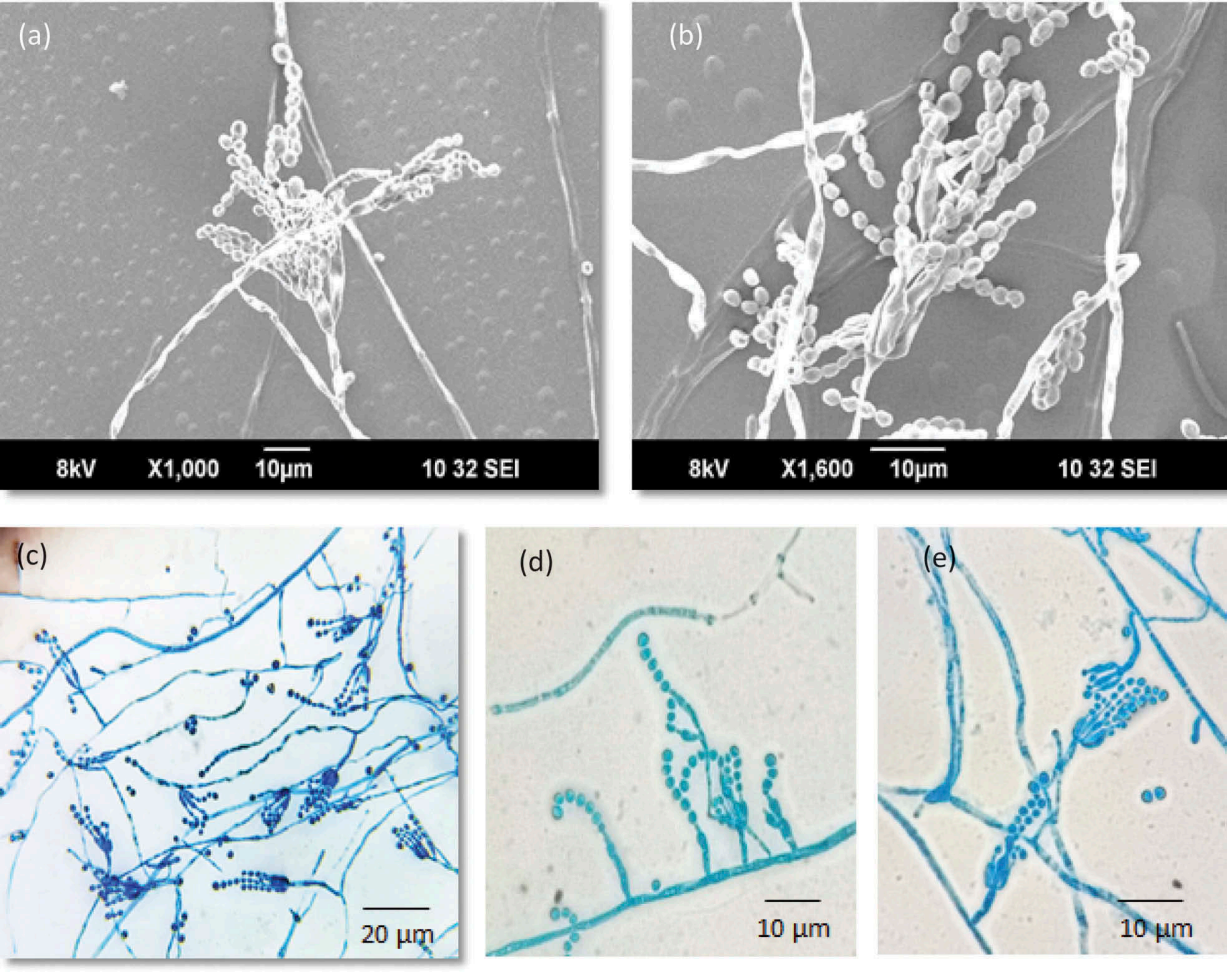
Conidiophores branching pattern of WSR 62: a, b. Scanning electron micrographs of conidiophore; c-e. Light microscopy: Short-to-moderate-sized conidiophores; solitary phialides and divaricate conidiophores.

**Figure 6.**
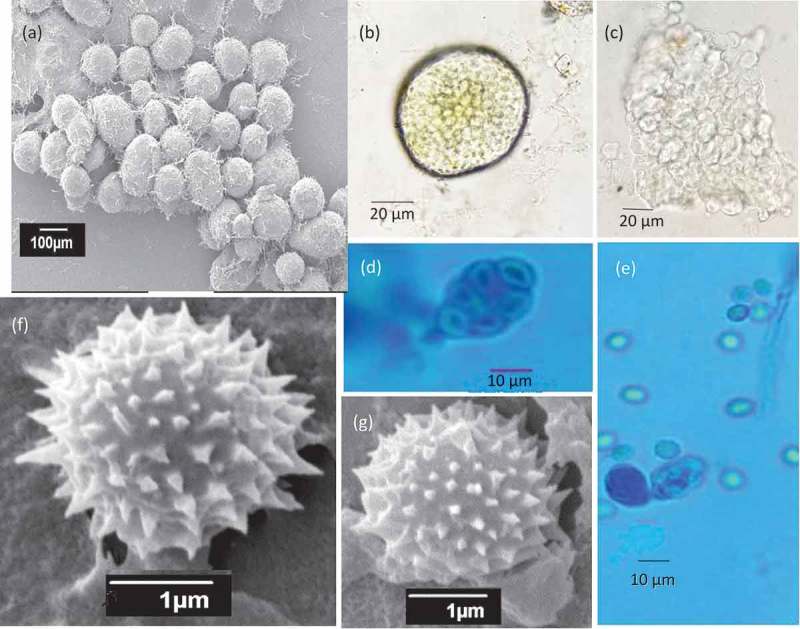
Morphological features of sexual reproduction stage of WSR 62. a. Cleistothecia; b, c. Ascospores inside the cleistothecia; d, e. Asci and ascospores released from the broken cleistothecia and ascus bearing eight ascospores; f, g. Ascospores with spines (SEM).

Conidiophores divaricate, few solitary phialides observed. Stipes smooth walled, variable in length, 10–50 × 2.0–3.0 μm, vesiculate, 2.0–3.5 µm; phialides 1–5 per stipe/metula, ampulliform, 5–7.5 × 2–3 μm. Conidia in long, loose, irregular columns, finely roughened, broadly ellipsoidal, some globose 2–3 × 2–2.5 μm (Figure 5). Cleistothecia globose to subglobose, abundantly produced on MEA, CYA and YES, 50–120 μm in diameter, solitary or clustered, maturing from centre to margins of the colony; surface covered with globose to hexagonal cells; outer 3–4 layers of cells are thin-walled, while the inner layer is brownish, with thick-walled cells. Asci 8-spored, pyriform when young, becoming subglobose to ellipsoidal at maturity, 8–12 × 5.0–8.5 μm. Ascospores globose to subglobose, 2.0–3.0 × 2.3–3.0 μm in diameter; hyaline to greenish yellow, spinose, spines measuring 0.2–0.4 μm (Figure 6).

#### Notes

A combination of features is unique for *Penicillium setosum*. This species has a greyish yellow obverse due to the abundant production of cleistothecia, and an orange brown reverse colony on CYA. *P. setosum* produces solitary phialides and divaricate type conidiophores with smooth-walled stipes. Furthermore, it produces small-sized (2.0–3.0 × 2.3–3.0 μm) globose to subglobose ascospores, decorated with spines measuring 0.2–0.4 μm.

## 4. Discussion

*Penicillium* section *Lanata*–*divaricata* is taxonomically challenging due to the morphological similarities among members of this section (Visagie et al. 2015). This taxonomically difficult section currently comprises 56 species (Visagie et al. 2016; Guevara-Suarez et al. 2017). Species belonging to section *Lanata*–*divaricata* are mainly soil inhabitants. Some species were isolated from the rhizosphere and phyllosphere of native plant communities, especially grasses and cereals, and some are frequently found on rotting leaf litter (Houbraken and Samson 2011; Visagie et al. 2015). In the present study, a novel endophytic *Penicillium* species was isolated from the surface of sterilized roots of *W. somnifera*. The two additional *P. setosum* strains were isolated from soil in Thailand and Mexico, showing the worldwide distribution of this species. There are other reports on endophytic species belonging to section *Lanata*–*divaricata. Penicillium janthinellum* was prominently reported as an endophyte from various plants such as *Melia azedarach* (Marinho et al. 2005), *Coffea arabica* (Vega et al. 2006), *Solanum lycopersicum* (Khan et al. 2013) and *Sonneratia caseolaris* (Zhu et al. 2017). Other endophytic species in section *Lanata-divaricata* are *P. oxalicum* from *Coffea* (Vega et al. 2006), *P. brasilianum* from *Melia azedarach* (Fill et al. 2010), *P. simplicissimum* and *P. javanicum* from maize and rice (Potshangbam et al. 2017).

The molecular study showed that *P. setosum* is most closely related with *P. javanicum*. Other species related to *P. setosum* are *P. elleniae, P. caperatum, P. malacosphaerulum*, and *P. reticulisporum*. All these species shared the ability to produce a sexual state. *Penicillium caperatum, P. malacosphaerulum,* and *P. reticulisporum* have roughened ascospores with two longitudinal flanges, *P. javanicum* has roughened ascospores and *P. elleniae* has broadly ellipsoidal ascospores with spinose valves (Visagie et al. 2015, 2016). These ascosporic structures differed from *P. setosum* which has spinose, globose to subglobose ascospores. The ascospores of *P. setosum* (2.0–3.0 × 2.3–3.0 μm) are smaller than those of *P. elleniae* (3–3.5 × 2.5–3) and *P. javanicum* (3–3.5 × 2–3). The length of the spines on the ascospores of *P. setosum* is comparable with that of *P. elleniae* (0.2–0.4 µm). Both *P. javanicum* and *P. setosum* abundantly produce cleistothecia on agar media. *Penicillium setosum* has a greyish yellow obverse colour and orange–brown reverse colony on CYA at 25°C, while *P. javanicum* has a light-brown obverse and a pale orange reverse and *P. elleniae* has yellow-brown (obverse and reverse) coloured colonies. *Penicillium setosum* grows faster on CYA than its close relatives. After 7 days incubation at 25°C, *P. setosum* has a colony diameter between 50 and 52 mm, while the colony diameters of the other species are (slightly) smaller (in mm): *P. javanicum* CBS 341.48^T^ (33–41), *P. malacosphaerulum* CBS 135120^T^ (28–36), *P. reticulisporum* CBS 122.68^T^ (45–46), *P. brefeldianum* CBS 235.81^T^ (36–38), *P. caperatum* CBS 443.75^T^ (38–40) and *P. elleniae* CBS 118135^T^ (48–50) (Visagie et al. 2015, 2016). Sporulation was sparse to absent in *P. setosum*, while *P. javanicum* showed abundant sporulation. Solitary phialides and divaricate type conidiophores with smooth-walled stipes were observed in *P. setosum*, while *P. javanicum* is reported to have rough-walled stipes and monoverticillate, or occasionally biverticillate penicilli (Stolk and Samson 1983).

The taxonomic novelty of WSR 62, a *Penicillium* isolate from the root of *Withania somnifera*, was determined using a polyphasic approach combining molecular data and phenotypic characters (e.g. colony colour and diameter, ornamentation of conidiophores and ascospore size, shape and ornamentation). Based on this polyphasic approach, we concluded that isolate WSR 62 represents a novel species, here named *Penicillium setosum* Tijith, Houbraken, Linu & Jisha.
